
JOURNAL INFORMATION
==============================
NLM Title Abbreviation: Wirtschaftsdienst
Journal ID: Wirtschaftsdienst
NLM Journal ID: 101086612

Wirtschaftsdienst (Hamburg, Germany : 1949)

ISSN: 0043-6275

ARTICLE INFORMATION
==============================
PMCID: PMC7174819
PMID: 32336807
DOI: 10.1007/s10273-020-2636-0
Article ID: 2636
Article version: 1
Subjects: Ökonomische Trends

Nachfrageorientierte Klimapolitik — Evidenz aus der Corona-Krise

Demand-oriented climate policy - Evidence from the corona crisis

Steinkamp Sven sven.steinkamp@uni-osnabrueck.de

grid.10854.38 0000 0001 0672 4366 Fachgebiet VWL - Intern. Wirtschaftspolitik, Universität Osnabrück, Rolandstraße 8, 49069 Osnabrück, Deutschland
Electronic publication date: 2020 Apr 22
Print publication date: 2020
Volume: 100
Issue: 4
First page: 300
Last page: 302
Copyright: © Der/die Autor(en) 2020
License: Open Access Dieser Artikel wird unter der Creative Commons Namensnennung 4.0 International Lizenz veröffentlicht, welche die Nutzung, Vervielfältigung, Bearbeitung, Verbreitung und Wiedergabe in jeglichem Medium und Format erlaubt, sofern Sie den/die ursprünglichen Autor(en) und die Quelle ordnungsgemäß nennen, einen Link zur Creative Commons Lizenz beifügen und angeben, ob Änderungen vorgenommen wurden.
Die in diesem Artikel enthaltenen Bilder und sonstiges Drittmaterial unterliegen ebenfalls der genannten Creative Commons Lizenz, sofern sich aus der Abbildungslegende nichts anderes ergibt. Sofern das betreffende Material nicht unter der genannten Creative Commons Lizenz steht und die betreffende Handlung nicht nach gesetzlichen Vorschriften erlaubt ist, ist für die oben aufgeführten Weiterverwendungen des Materials die Einwilligung des jeweiligen Rechteinhabers einzuholen.
Weitere Details zur Lizenz entnehmen Sie bitte der Lizenzinformation auf http://creativecommons.org/licenses/by/4.0/deed.de.
License URL: https://creativecommons.org/licenses/by/4.0/

issue-copyright-statement: © The Author(s) 2020

==============================

Der Klimawandel ist ohne Zweifel eine der größten globalen Herausforderungen für die Wirtschaftspolitik. Nur bei wenigen Fragestellungen ist die Diagnose so klar, der Handlungsdruck so akut. Gemein haben die meisten bisher ergriffenen Maßnahmen, dass sie versuchen, dem Klimawandel durch Reduktion der nationalen Nachfrage nach fossilen Brennstoffen zu begegnen. Ein aktuelles Beispiel ist die „Unter 1000 mach ich‘s nicht“-Kampagne der „Scientist for Future“-Bewegung. Rund 2000 Professorinnen und Professoren hatten sich kurz vor der Corona-Krise selbstverpflichtet, bei Dienstreisen unter 1000 km auf Flüge zu verzichten. Wie groß kann die Wirkung solcher Initiativen sein?

Es ist bei makroökonomischen Fragen immer schwierig, kausale Wirkungszusammenhänge in den Daten zu erkennen, da sowohl Angebot als auch Nachfrage den Preis beeinflussen und man nur selten eine Seite isoliert betrachten kann. Ereignisse wie das Corona-Virus eröffnen im Fall der Klimapolitik aber eine klarere Sicht. Das Virus verursacht einen exogenen, regionalen Öl-Nachfrageschock. Dieser wirft nicht nur ein Licht auf obige Frage, sondern lässt viel allgemeiner Schlüsse auf die Wirksamkeit nachfrageorientierter Klimapolitik zu.

Nationale Klimapolitik, mit dem Ziel einer Reduktion der Nachfrage nach fossilen Brennstoffen, ist alles andere als unstrittig, denn vielleicht verschiebt sich dadurch lediglich der Verbrauch in andere Regionen der Welt. Die Effekte auf die $CO_2$-Bilanz gleichen sich dann gerade aus, die Maßnahme ist wirkungslos. Aus ressourcenökonomischer Sicht ist die Gesamtmenge an Erdöl begrenzt. Sofern die Fördermengen sich nicht ändern, führt jede regionale Einsparung, z. B. in Deutschland, zu einer Preisänderung bei Erdöl – nicht aber zu einer Mengenänderung, die für das Klima notwendig wäre. Das Öl wird nur an anderer Stelle verbrannt (Sinn, 2012).

Doch woher weiß der Rest der Welt, dass in Deutschland fossile Brennstoffe gespart werden und sie nun im Gegenzug mehr konsumieren können? Kein zentrales Gremium koordiniert den Ölmarkt. Nein, es ist die berühmte unsichtbare Hand von Adam Smith. Der Preis koordiniert Anbieter und Nachfrager und räumt den Markt. Der Verzicht Deutschlands lässt die globalen Preise fallen und koordiniert das fixe globale Angebot mit der gesunkenen regionalen Nachfrage. Die Ersparnis in Deutschland wirkt wie eine Subvention auf den Ölverbrauch anderer Länder.

Das Corona-Virus verursacht eine von Preis und Angebot unbeeinflusste Nachfrageänderung nach Öl, auch weil seit Anfang des Jahres ein erheblicher Teil des Flugverkehrs zum Erliegen gekommen ist. Sicherlich ist dies im Hinblick auf die Ölnachfrage ein größeres Ereignis als die Selbstverpflichtung deutscher Wissenschaftler. Neben zehntausenden Flugstreichungen, tun regionale Reiserestriktionen und die Absage von Großveranstaltungen ihr Übriges. Bei aller Gefahr des Virus – getreu der Grundannahme nachfrageorientierter Wirtschaftspolitik müsste man vermuten, dass dies zumindest eine Entspannung der $CO_2$-Bilanz zur Folge hätte.

## Wie reagieren Ölpreis und -produktion auf regionale Nachfrageschocks?

Die kleine Ereignisstudie in Abbildung 1a veranschaulicht die Entwicklung der Daten nach Ausbruch der Corona-Krise. Seit der Ankündigung der Weltgesundheitsorganisation (WHO) am 6.1.2020, ein neues Virus der Corona-Gruppe (SARS-CoV-2) sorge möglicherweise für eine weltweite Pandemie, sind die Rohölpreise im freien Fall. Die Preise sind von ca. 70 Euro pro Barrel auf 32 Euro pro Barrel (-45 %) bis Mitte März 2020 gefallen.[1] Eine signifikante Änderung der Fördermenge blieb bis dato jedoch aus. Allerdings gibt es bisher auch nur zwei Monate (Januar und Februar 2020), die man zur Beurteilung heranziehen könnte, denn die weltweiten Produktionsmengen werden nur monatlich erhoben.

1 Rohölpreis (Brent Europa), 12. März 2020.

Abbildung 1
**Ereignisstudie zur globalen Ölproduktion**

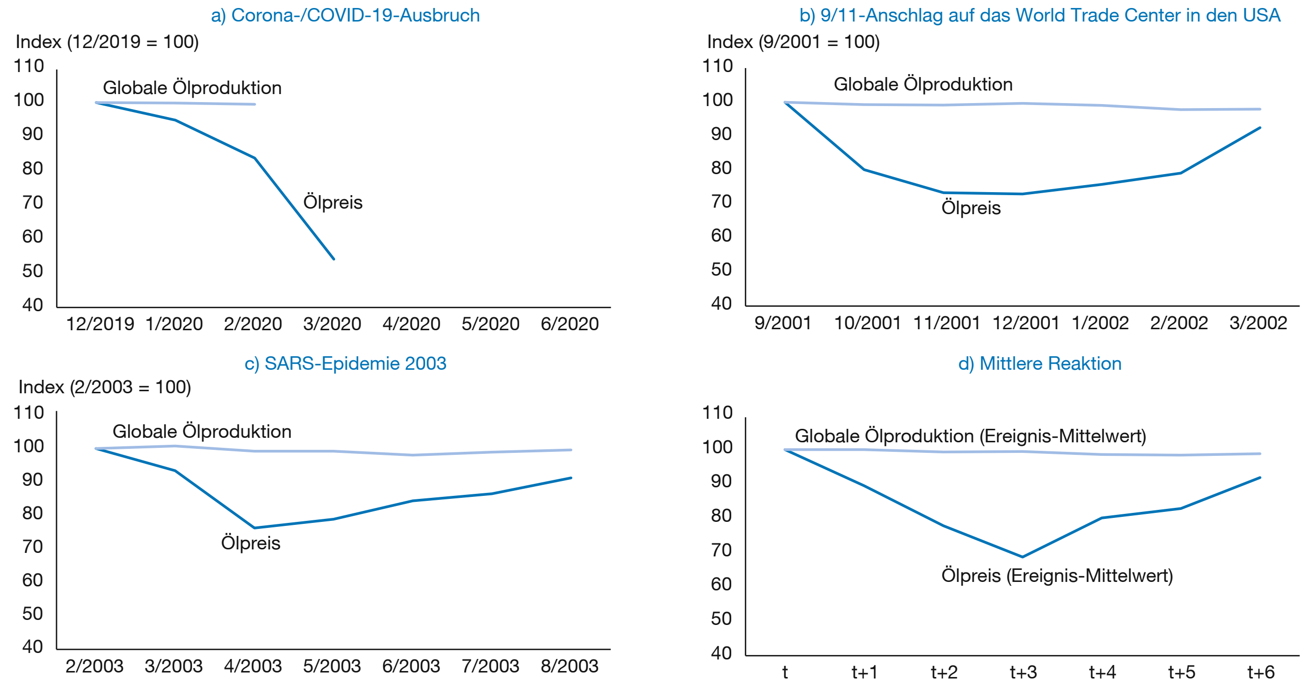

Anmerkung: Die Daten sind jeweils auf den Ereignismonat normiert. Der Datenpunkt März 2020 bezieht sich auf den 12.3.2020.

Quellen: Federal Reserve Bank of St. Louis; US Energy Information Administration; OPEC.

Nicht viel anders war die Marktreaktion nach den Anschlägen am 11. September 2001 in den USA (vgl. Abbildung 1b) oder auch bei der SARS-Epidemie von 2003 (vgl. Abbildung 1c), als beide Male ebenfalls der Flugverkehr regional eingebrochen ist. Der statistische Vorteil dieser Ereignisse ist, dass auch Daten über den direktbetroffenen Zeitraum hinaus vorliegen. Erneut sieht man: Kurz nach den Ereignissen fielen die Erdölpreise um mehr als 20 %, aber die globale Fördermenge blieb davon fast unbeeinträchtigt. Vergleicht man alle drei Fälle oder betrachtet die mittlere Reaktion (vgl. Abbildung 1d), ist es erstaunlich, wie stabil das Ölangebot ist.

Natürlich hat ein narrativer Analyseansatz wie dieser Grenzen. Es könnte beispielsweise sein, dass einmalige und kurzfristige Änderungen in der Nachfrage keine Wirkung auf die Fördermenge haben, viele kleine Einsparungen über einen längeren Zeitraum hingegen schon. Aber auch hier besteht kein Grund zu Optimismus. Bereits vor zehn Jahren zeigte Ressourcenökonom Lutz Kilian überzeugend, dass die von 1976 bis 2009 identifizierten Ölnachfrageschocks keinen nennenswerten Einfluss auf die Produktionsmenge hatten, wohl aber auf die Preise (Kilian, 2009). Ein Ergebnis, das nun durch die Corona-Krise – mit viel weniger ökonometrischem Aufwand – bestätigt wird.

Vielleicht wird das geförderte Öl aber eingelagert statt verbrannt. Zwei Fakten sprechen dagegen: Erstens erfreuen sich Terminkontrakte zum Recht auf Einlagerung von Erdöl (LOOP Crude Oil Storage Futures) derzeit keiner großen Beliebtheit und werden kaum gehandelt. Zweitens hat die Einlagerung zeitliche Grenzen. Zumindest in der langen Sicht ist anzunehmen, dass Ereignisse (z. B. Klimaabkommen, Emissionshandelssysteme) in der Vergangenheit Einfluss auf die Öl-Produktionsmengen genommen hätten.

Abbildung 2 zeigt hingegen, dass dies offenbar nicht der Fall ist. Die Produktionsmengen haben einen leichten, nahezu linearen Trend nach oben. Vergleicht man die mittelwertbereinigte Reihe des Ölpreises mit der Produktionsmenge, wird klar, dass die Variation des Ölpreises deutlich ausgeprägter ist. Während dessen Variationskoeffizient 0,70 beträgt, ist er bei der globalen Produktionsmenge lediglich 0,11. Dies ist prima facie Evidenz für die kurzfristige Inelastizität des Öl-Angebotes.

## Kurze versus lange Sichtweise

Wenn regionale Nachfrageschocks die Fördermenge unberührt lassen, stellt sich die Frage, was dessen Auf-

Abbildung 2

**Ölpreis und -produktionsmenge – eine 30-Jahres-Perspektive**

mittelwertbereinigt

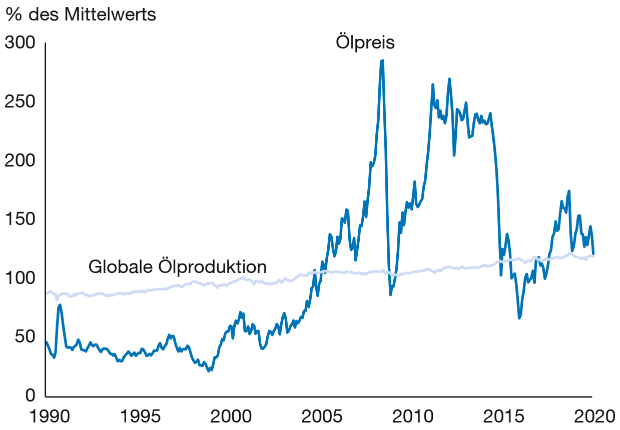

Quellen: Federal Reserve Bank of St. Louis; US Energy Information Administration; OPEC.

Tabelle 1

**Korrelationen der Trend- und Zykluskomponenten**

| Zyklus | Reale Wirtschaftstätigkeit | Ölangebot |
|---|---|---|
| Ölangebot | 0,032<br>(0,5323) | – |
| Ölpreis | 0,572<br>(0,0000) | 0,109<br>(0,0387) |
| Trend | | |
| Ölangebot | 0,964<br>(0,0000) | – |
| Ölpreis | 0,823<br>(0,0000) | 0,740<br>(0,0000) |

Anmerkung: Die Tabelle zeigt bivariate Korrelationskoeffizienten und t-Test-Signifikanzniveaus der Trend- und Zykluskomponenten des Ölpreises, der Ölproduktion und der realen Wirtschaftsaktivität. Letztere wird gemessen durch die Stahlproduktion. Vgl. Ravazzolo, F. und J. L. Vespignani (2020), A New Monthly Indicator of Global Real Economic Activity, *Canadian Journal of Economics*, im Erscheinen 2020. Trend-Zyklus-Zerlegung mittels Hodrick-Prescott-Filter.

Quellen: Federal Reserve Bank of St. Louis; US Energy Information Administration; OPEC; World Steel Association.

wärtstrend treibt. Ein naheliegender Kandidat ist dabei der langfristige Anstieg der globalen Wirtschaftstätigkeit. Deutlich erkennen lässt sich dies, wenn man zunächst alle drei Reihen in ihren Zeittrend und ihre zyklische Komponente zerlegt, und dann die Korrelationskoeffizienten vergleicht.

Tabelle 1 zeigt, dass es keine nennenswerte Korrelation zwischen den Schwankungen der realen Wirtschaftstätigkeit und der Ölförderung gibt. Schwankungen des Ölpreises hingegen gehen deutlich mit Änderungen der globalen Produktionstätigkeit einher. Der Korrelationskoeffizient beider (stationärer) Zeitreihen beträgt 0,55 und ist statistisch signifikant.

Anders sieht es bei dem langfristigen Trend der Reihen aus. Hier gibt es erwartungsgemäß hohe, statistisch-signifikante Korrelationen zwischen dem Ölpreis, der Fördermenge und der Wirtschaftstätigkeit. Angebot, Nachfrage und Preis stehen in einer stabilen Langfristbeziehung zueinander.[2]

## Wirtschaftspolitische Implikationen

Die Schlussfolgerung ist daher explizit nicht, dass eine globale, langfristige Nachfragereduktion nach fossilen Brennstoffen nicht notwendig oder effektiv wäre. Es steht auch außer Frage, dass der Klimawandel ein zentrales Problem ist.

Die Analyse holt aber vielleicht diejenigen zu einer ernüchternden Sicht zurück, die glauben, dass ein nationaler Alleingang bei nachfrageseitiger Klimapolitik ausreicht. Zumindest kurzfristig – und dies ist in Anbetracht selbst optimistischer Klimaprognosen der relevante Handlungshorizont – ist die unsichtbare Hand dafür zu stark. Bleibt der Fokus auf nachfrageorientierter Klimapolitik, muss diese global und langfristig sein, also in einen starken multilateralen Koordinationsrahmen eingebettet werden. Gelingt dies nicht, muss angebotsseitige Klimapolitik mehr Raum bekommen.[3]

2 Das bekannte Problem der Interpretation nicht-stationärer Variablen sei nicht verschwiegen. Ökonometrisch bestätigt wird der gemeinsame Langfristtrend jedoch ebenfalls durch Johansen-Kointegrationstests sowohl für den bivariaten als auch den trivariaten Fall.

3 Dass letzteres auch national wirksam sein kann, zeigen Gerarden, Spencer Reeder und Stock (2020).

Dr. Sven Steinkamp ist Akademischer Rat an der Universität Osnabrïck.


Literatur

Gerarden T D Spencer Reeder W Stock J H Federal Coal Program Reform, the Clean Power Plan, and the Interaction of Upstream and Downstream Climate Policies American Economic Journal: Economic Policy 2020 12 1 167 199
Kilian L Not All Oil Price Shocks Are Alike: Disentangling Demand and Supply Shocks in the Crude Oil Market American Economic Review 2009 2009 99 3 1053 1069
Ravazzolo F Vespignani J L A New Monthly Indicator of Global Real Economic Activity Canadian Journal of Economics 2020
Sinn H-W The green paradox: a supply-side approach to global warming 2012
